# 20-year experience on prenatal diagnosis in a reference university medical genetics center in Turkey

**DOI:** 10.3906/sag-2006-103

**Published:** 2021-08-30

**Authors:** Burak DURMAZ, Hilmi BOLAT, Zehra CENGİSİZ, Fuat AKERCAN, Tuba SÖZEN TÜRK, Erhan PARILTAY, Aslı Ece SOLMAZ, Mert KAZANDI, Emin KARACA, Asude DURMAZ, Ayça AYKUT, Sermet SAĞOL, Haluk AKIN, Ferda ÖZKINAY, Özgür ÇOĞULU

**Affiliations:** 1 Department of Medical Genetics, Faculty of Medicine, Ege University, İzmir Turkey; 2 Division of Perinatology, Department of Obstetrics and Gynecology, Faculty of Medicine, Ege University, İzmir Turkey; 3 Division of Genetics, Department of Pediatrics, Faculty of Medicine, Ege University, İzmir Turkey

**Keywords:** Prenatal diagnosis, chromosome abnormalities, genetic counseling

## Abstract

**Background/aim:**

Although cutting edge procedures such as cell-free fetal DNA isolation from maternal blood are now available, invasive prenatal tests are still being used extensively for prenatal diagnosis. The study aims to evaluate the demographic data, indications, and cytogenetic results of 9297 results of patients who underwent prenatal invasive testing for genetic analysis that were referred for the last 20 years in a University Medical Genetics Center.

**Materials and methods:**

The records of 8363 amniocenteses, 626 chorionic villus, and 308 cordocenteses samples were retrospectively evaluated and analyzed regarding referral reasons, indications and their cytogenetic results. The total numbers and the percentages of each group were recorded; Chi-square and logistic regression analyses were performed to give the statistical likelihood of different events.

**Results:**

The number of referrals decreased signiﬁcantly after 2009. Risk of having trisomy 21 as well as trisomy 13 and 18 significantly increased in parallel with advanced maternal age. When the 21–25 age group was compared to the older age groups in terms of having a trisomy 21 pregnancy, the risk doubled in the 36–40, 5 times higher in 41–45 and 10-fold in 46–50 age groups. No significant linear correlation between maternal serum screening test results and trisomy 21 was found, however the difference between the pregnancies whom cut-off value above and below 1/250 in maternal serum screening test were significant.

**Conclusion:**

These data have provided useful information on the frequency of referrals to the reference genetics department, and the feasibility of genetic services. By reviewing the indications and their corresponding results, we can offer invaluable insights that will be useful in genetic counseling and also in the development of more effective genetic strategies.

## 1. Introduction

Prenatal screening and diagnostic testing for chromosomal abnormalities have expanded dramatically over the past two decades [1]. After reviewing 68,159 live born baby surveys from 1969 to 1982, 1 in 156 live births were found to be carrying a major chromosomal abnormality [2]. The implementation of effective screening tests decreased the need for invasive diagnostic testing such as amniocentesis (AC), chorionic-villus sampling (CVS) or cordocentesis; thus, reducing the risk of procedure-related miscarriages for a healthy pregnancy. Screening tests, however, have limitations which include both false positive and false negative results and currently cannot detect all chromosomal anomalies.

While more advanced procedures such as cell-free fetal DNA (cffDNA) testing from maternal blood are available, invasive prenatal tests are still being used extensively for prenatal diagnosis in most countries. Amniocentesis and CVS are considered safe and accurate procedures. The risk of major complication associated with midtrimester amniocentesis at 15–16 weeks of gestation is 1 in 1600. The risk of inducing miscarriage is only approximately 1%–2% over the baseline risk for any pregnancy at this stage of gestation [3]. The decision regarding screening to mitigate the risk having a child with chromosomal abnormality prior to the child’s birth is very personal and deeply complex. The presence of a family history of aneuploidy or genetic disorders, obstetrical and medical history, demographic information, attitudes and religious beliefs, education levels and economic concerns are all influencing factors [4]. Investigation of these factors may offer invaluable insights that will be used in genetic counseling services prior to prenatal diagnosis, and also in the development of more effective decision-making strategies. Therefore, the aim of this study was to evaluate and to discuss the demographic data, indications and cytogenetic results of 9297 cases that opted for prenatal sampling procedures.

## 2. Materials and methods 

The study was conducted retrospectively using the records of AC, CVS, and cordocentesis referrals for the last 20 years in a University Medical Genetics Center. A total of 9297 women between 18–54 years old were included in the study. Invasive prenatal testing was performed for each case appropriate to its week of gestation (CVS at 10–13 weeks of gestation, AC at 16–22 weeks of gestation, and cordocentesis at 20–24 weeks of gestation). In all cases, genetic counseling was provided, outlining the chances of finding chromosomal abnormality in the fetus and the risks associated with invasive prenatal testing procedures. Invasive prenatal testing was performed only after written informed consent had been obtained. 

We grouped the cases according to the standard indications of prenatal testing (Table 1):

**Table 1:** Number of chromosomal abnormalities detected in regard to the indications of prenatal diagnosis.

Indications	Number of total cases (%)	Number of abnormal karyotype with inv(9) (%)	Number of abnormalkaryotype without inv(9) (%)	Positive predictive value (%)
Advanced maternal age	4482 (48.2)	205 (4.5)	160 (3.5)	3.5
Increased risk in MSS tests	2387 (25.7)	107 (4.4)	69 (2.8)	2.8
Abnormal ultrasound findings	1021 (11)	121 (11)	101 (9.8)	9.8
Referral for molecular analysis	695 (7.5)	14 (2)	5 (0.7)	0.7
Previous pregnancy with chromosome abnormality	249 (2.7)	19 (7.8)	18 (7.2)	7.2
Other indications*	172 (1.8)	6 (3.4)	4 (2.3)	2.3
Advanced maternal age with abnormal ultrasound findings	106 (1.1)	30 (28)	29 (27.3)	27.3
Maternal anxiety	86 (0.9)	5 (5.8)	4 (4.6)	4.6
Parental abnormal karyotype	41 (0.4)	15 (36.5)	15 (36.5)	36.5
Increased risk in MSS tests with abnormal ultrasound findings	26 (0.3)	4 (15.3)	4 (15.3)	15.3
Recurrent pregnancy loss	21 (0.2)	3 (14.2)	3 (14.2)	14.2
Previous pregnancy with chromosome abnormality and advanced maternal age	11 (0.1)	9 (81.8)	9 (81.8)	81.8
Total	9297 (100)	538	421	-

1. Advanced maternal age (AMA) (≥35 years at the expected time of delivery)

2. Abnormal result of maternal serum screening tests

3. Abnormal ultrasound (USG) ﬁndings

4. Referral for molecular analysis

5. Chromosome abnormality in previous pregnancy

6. Advanced maternal age with abnormal ultrasound findings

7. Parental anxiety

8. Abnormal parental karyotype

9. Chromosome abnormality in previous pregnancy with advanced maternal age

10. Recurrent pregnancy loss

11. Other indications

Cases with both AMA and abnormal USG finding, or AMA and chromosome abnormality in previous pregnancy were grouped separately. 

Cases were further classified into groups according to age: 

1. 21–25 years

2. 26–30 years

3. 31–35 years

4. 36–40 years 

5. 41–45 years 

6. 46–54 years

A positive cytogenetic result was accepted if the obtained karyotype was other than 46,XX or 46,XY and these results were grouped as numerical or structural abnormality. The study was approved by Ege University Scientific Research Ethics Committee with the approval number 17-8.1/11.

### 2.1. Statistical analyses

The total number of events and the percentages of each group were recorded. Chi-square and logistic regression analysis were performed in order to give the statistical likelihood of different events. A p-value of < 0.05 was considered statistically significant. Positive predictive values were calculated for each of the prenatal indications with the cytogenetic results. The Shapiro–Wilk test was employed to verify the normality of the data. The data generated from the study were compared and analyzed using SPSS v: 19.0 software (IBM Corp., Armonk, NY, USA) with confidence interval set at 95%.

## 3. Results

Analyses were carried out on 9297 prenatal sample results; AC (8363 cases), CVS (626 cases), and cordocentesis (308 cases). The Shapiro–Wilk test revealed normal distribution of the data (p > 0.05). Our culture success rate was 98.4%. The rate of chromosomal abnormalities was found to be 4.5% excluding inversion 9 (inv(9)) polymorphism being trisomy 21 was the most common cytogenetic abnormality. Mean age for all pregnancies was 33.17±5.9; ranging between 18–54 years, whereas 50.8% of cases were advanced maternal age (≥35 years of age). The mean gestational duration of the pregnancies at the time of testing was 17.4**±**2.2 weeks. It is interesting to note that the number of referrals decreased signiﬁcantly after 2009.

By volume, the indications for prenatal diagnosis were AMA in 4482 cases (48.2%), abnormal maternal screening tests in 2387 cases (25.7%), abnormal USG ﬁndings in 1021 cases (11.1%), referral for molecular analysis in 695 cases (7.5%), and chromosome abnormality of a previous pregnancy in 249 cases (2.7%). Further indications included AMA with abnormal ultrasound finding in 106 cases (1.1%), parental anxiety in 86 cases (0.9%), having abnormal parental karyotype in 41 cases (0.4%), recurrent pregnancy loss in 21 cases (0.2%), abnormality in previous pregnancy with AMA in 11 cases (0.1%), other indications including stillbirth in previous pregnancy, failure in previous prenatal test, which accounted for 172 cases, (1.8%). Table 1 summarizes the detected chromosomal abnormalities according to the indications of prenatal diagnosis.

Of the 9297 cases, chromosome anomalies and inv(9) polymorphism were determined in 538 cases (5.8%). Numerical abnormalities accounted for 60.1% and structural abnormalities made up the remaining 39%. Of the numerical chromosomal abnormalities, trisomy 21 was noted in 167 cases (31%), an abnormality of sex chromosome in 57 cases (10.6%), trisomy 18 in 42 cases (7.8%), marker chromosome in 18 cases (3.3%), triploidy or tetraploidy in 16 cases (3%), trisomy 13 in 13 cases (2.4%), and mosaic chromosomal abnormalities were noted in 11 cases (2%). With reference to the structural anomalies, 64 cases had translocation (11.9%), in which 45 patients had reciprocal, and 19 patients had Robertsonian translocation, 19 cases of inversion (3.5%), and 10 cases of deletion (1.9%). Rare structural anomalies were reported in 4 cases in which 3 cases had derivative chromosome and 1 case had isochromosome X (0.7%). Additionally, inv(9) polymorphism was found in 117 cases (21.7%). Chromosome analysis was recommended from the parents of patients having structural chromosomal anomalies. The outcomes of 154 patients, whose records have been reached, whose parents were alive and who wanted to have the karyotype analysis were evaluated. Segregation analysis revealed that, 24 reciprocal translocations, 12 Robertsonian translocations, 13 inversions, 3 derivative chromosomes, 1 deletion, and 87 inv(9) polymorphisms were segregated from either parent and noted as familial. On the other hand, 5 reciprocal translocations, 2 Robertsonian translocations, 6 deletions, and 1 isochromosome were de novo. Table 2 summarizes the distribution of numerical and structural chromosomal anomalies.

**Table 2:** Distribution of numerical and structural chromosomal abnormalities.

Chromosomal abnormalities	Number (%)	
Numerical	324 (60.1)	
Trisomy 21	167 (31.0%)	
Sex chromosome abnormality	57 (10.6%)	
Trisomy 18	42 (7.8%)	
Marker chromosome	18 (3.3%)	
Triploidy/tetraploidy	16 (3.0%)	
Trisomy 13	13 (2.4%)	
Mosaic chromosomal abnormality	11 (2%)	
Structural	214 (39.0%)	Familial/De-novon (%)/n (%)
Translocation	64 (11.9%)	36 (23.4%)/7 (4.5%)
Inversion	19 (3.5%)	13 (8.4%)/0 (0%)
Deletion	10 (1.9%)	1 (0.6%)/6 (3.9%)
Rare chromosomal abnormality*	4 (0.7%)	3 (1.9%)/1 (0.6%)
Inv(9) polymorphism	117 (21%)	87 (56.5%)/0 (0%)
Total	538 (100%)	154 (100%)

*Rare chromosomal abnormality (derivative chromosome abnormality and isochromosome) (Inv: inversion, n: number).

The indication of “chromosome abnormality in previous pregnancy with advanced maternal age” was found to be the most strongly associated with chromosomal abnormalities (81.8%). A positive predictive value (PPV) was also detected in other indications such as parental abnormal karyotype (36.5%), advanced maternal age with abnormal ultrasound findings (27.3%), increased risk in MSS tests with abnormal ultrasound findings (15.3%), recurrent pregnancy loss (14.2%), abnormal ultrasound findings (9.8%), previous pregnancy with chromosome abnormality (7.2%), maternal anxiety (4.6%), AMA (3.5%), increased risk in MSS tests (2.8%), and others (2.3%). The indication “referral for molecular analysis” produced a PPV of only 0.7% (Table 1).

Logistic regression analysis revealed a strong correlation with the risk of having trisomy 21 and AMA (2.6%, p < 0.001). However, while the risk increased for AMA pregnancies in terms of trisomy 18 and 13, the diﬀerence was not statistically signiﬁcant in Chi-square test (p > 0.05). The risk of having a trisomy 21 pregnancy was then evaluated across diﬀerent age groups with logistic regression analysis. When the 21–25 age group was compared to the older age groups, risk doubled in the 36–40 age group. This was 5 times and 10 times higher in the 41–45 and 46–50 age groups, respectively. Table 3 summarizes the risk of having a child with Down Syndrome according to the different age groups.

**Table 3:** Risk of having a child with Down Syndrome (DS) compared to age range of 21–25.

Age	DS risk (X)	B	S.E	OR	%95 C.I.	p
26–30	1	0.143	0.389	1.154	0.538–2.475	0.713
31–35	1	0.120	0.372	1.127	0.543–2.337	0.748
36–40	2	0.806	0.339	2.238	1.151–4.351	0.018*
41–45	5	1.669	0.363	5.305	2.603–10.814	<0.001*
46–54	10	2.285	684	9.83	2.573–37.555	0.001*

A total of 2387 cases were referred due to increased risk in maternal serum screening. We were able to access the detailed data of 1747 cases. In 1497 cases, the risk was below 1/250 (high risk), and in 250 cases, the risk was 1/250 or higher (low risk). We were able to establish a cut-oﬀ value of 1/250 for trisomy 21. However, Chi-square test was unable to establish any signiﬁcant correlation between maternal serum screening test results higher than 1/250 and trisomy 21 occurrence (p > 0.05). All the pregnancies that were found to have trisomy 21 also had an increased risk in maternal serum screening which is below 1/250 cut-off value. 

## 4. Discussion

Genetic counseling is an essential step prior to prenatal testing and plays a very important role in meeting the expectations of families about prenatal screening tests. Understanding the risks and the likelihood ratio of detecting chromosomal abnormalities when these procedures were applied, have become very important aspects of the pretest genetic counseling [5]. In this study, chromosomal abnormalities has been reported in 4.5% of all cases with the exception of inv(9) polymorphism which is higher than the previous reports [4,6–8]. AMA is a well-known risk factor for chromosomal abnormality [6–11]. The detection rate of abnormal karyotype in women over ≥35 years old was higher than that previously reported by Xiao et al. (2.79%) [8]. However, it is similar to the study reported by Balkan et al. (4.9%) [12].

 In our study, the main indication for prenatal testing was AMA and the second indication was the presence of abnormal MSS tests. In recent years, particularly in developed countries, the mean age of pregnant women has increased [13]. In previous studies, while the most common indications for prenatal testing were AMA [14,15] and MSS [9], with the increased development of MSS procedures, chromosomal abnormality detection rates have increased to higher rates. This caused MSS tests to become a more frequent indication for an invasive testing [16]. In a recent retrospective study, it was shown that there was a significant change in the trend of indications for invasive prenatal diagnosis from advanced maternal age in 2009 to positive screening tests by 2014 [17]. In a study from Turkey, highest chromosomal anomaly detection rate was observed in pregnant women with increased risk in MSS (3.2%) [18]. Although the use of noninvasive maternal next generation screening tests is gradually increasing, their sensitivity and specificity should be questioned. Therefore, the meaning of the rates determined in traditional screening tests should also be well explained in genetic counseling sessions. In our study, there was no increase in the rate of chromosomal anomaly detection rate in parallel with the rate determined in MSS. However, trisomy 21 detection rate was significantly higher in women having a MSS test above 1/250 cut-off value [18]. An important aspect of prenatal care has always been the consideration of trisomy 21. In our study, the risk of having a trisomy 21 pregnancy was also evaluated across diﬀerent age groups. When the 21–25 age group was compared to the older age groups, risk doubled in the 36–40 age group. This was found to be 5 and 10 times increased in 41–45 and 46–50 age groups, respectively. However, we have not found any significant association between AMA and trisomy 13 or trisomy 18.

The indication of “Chromosome abnormality in previous pregnancy with advanced maternal age” was found to be the indication, most strongly associated with any chromosomal abnormality (81.8%). It was considered to be a high indicator of an abnormal karyotype; therefore, the cytogenetic results should be thoroughly reviewed for any subtle chromosomal abnormality. “Fetal anomalies in prenatal ultrasonography” was the most common indication in some other studies associated with chromosomal anomalies which ranged between 5.3%–20.3% [14–15,19–21]. Our PPV value for abnormal USG findings was 9.8% which was similar to previous results. Moreover, accompanying indications such as “advanced maternal age” or “abnormal MSS tests” in conjunction with abnormal USG findings increased the PPV value. Advanced maternal age with abnormal USG findings produced a rate of 27.3%, whilst abnormal MSS tests together with abnormal ultrasound findings had a rate of 15.3%. In a recent study by Sun et al. demonstrated that ultrasound soft markers were the most common prenatal diagnostic indication among 3387 patients, but the detection rate of abnormal karyotypes was only 2.02%. Additionally, it was 46.97% in the genome-wide NIPT-positive group which they concluded that NIPT should not be recommended as the first-tier screening for chromosomal aberration for pregnancies with ultrasound soft markers or pathological ultrasound findings [22]. 

There is an increase in the demand for prenatal tests due to lower costs and availability particularly after 2003. Amniocentesis is the most preferred prenatal procedure as, a 5-year retrospective study showed that, 86% of amniocentesis were safe and free from any complications [23]. Although the range and quality of prenatal tests have increased over the years, the number of requests for these tests in our department has declined since 2009. One possible explanation could be the decentralization of specialized services. The fact that these tests can now be carried out in more provincial hospitals may explain the decline since 2009. A cheaper, faster, and less intrusive option of USG imaging and combined screening tests together with noninvasive next generation prenatal screening may have also contributed to this decline.

Moreover, our culture success rate was 98.4%, which was around in the level of upper range in the studies reported from our country. In one of those studies, a cytogenetic result could be obtained in 98.8% of the 6124 cases evaluated by using AC [24]. In another study the culture success rate was 99% [25]. However, these studies reported only amniocentesis data. To gain a broader perspective, our study included all forms of prenatal sampling procedures.

## 5. Conclusion

As technology evolves, the opportunity for early diagnosis of genetic diseases has become a very useful addition to the healthcare system. We think that, by reviewing these indications and their corresponding results, it provides a helpful insight for genetic counseling prior prenatal diagnosis and also adds useful background to the strategic development of effective and long-term genetic services.
